# CpG island methylation profile in non-invasive oral rinse samples is predictive of oral and pharyngeal carcinoma

**DOI:** 10.1186/s13148-015-0160-7

**Published:** 2015-12-03

**Authors:** Scott M. Langevin, Melissa Eliot, Rondi A. Butler, Agnes Cheong, Xiang Zhang, Michael D. McClean, Devin C. Koestler, Karl T. Kelsey

**Affiliations:** Department of Environmental Health, University of Cincinnati College of Medicine, 160 Panzeca Way, ML0056, Cincinnati, OH 45267 USA; Department of Epidemiology, Brown University, Providence, RI USA; Department of Veterinary and Animal Sciences, University of Massachusetts Amherst, Amherst, MA USA; Department of Environmental Health, Boston University School of Public Health, Boston, MA USA; Department of Biostatistics, University of Kansas Medical Center, Kansas City, KA USA; Department of Pathology and Laboratory Medicine, Brown University, 70 Ship Street, Box G-E3, Providence, RI 02912 USA

**Keywords:** Head and neck cancer, DNA methylation, Biomarkers, Infinium, Mouthwash

## Abstract

**Background:**

There are currently no screening tests in routine use for oral and pharyngeal cancer beyond visual inspection and palpation, which are provided on an opportunistic basis, indicating a need for development of novel methods for early detection, particularly in high-risk populations. We sought to address this need through comprehensive interrogation of CpG island methylation in oral rinse samples.

**Methods:**

We used the Infinium HumanMethylation450 BeadArray to interrogate DNA methylation in oral rinse samples collected from 154 patients with incident oral or pharyngeal carcinoma prior to treatment and 72 cancer-free control subjects. Subjects were randomly allocated to either a *training* or a *testing* set. For each subject, average methylation was calculated for each CpG island represented on the array. We applied a semi-supervised recursively partitioned mixture model to the CpG island methylation data to identify a classifier for prediction of case status in the *training* set. We then applied the resultant classifier to the *testing* set for validation and to assess the predictive accuracy.

**Results:**

We identified a methylation classifier comprised of 22 CpG islands, which predicted oral and pharyngeal carcinoma with a high degree of accuracy (AUC = 0.92, 95 % CI 0.86, 0.98).

**Conclusions:**

This novel methylation panel is a strong predictor of oral and pharyngeal carcinoma case status in oral rinse samples and may have utility in early detection and post-treatment follow-up.

## Background

Oral and pharyngeal cancer are major public health concerns in the USA, where there were an estimated 42,440 new cases of oral and pharyngeal cancer diagnoses (it is the eighth most common form of cancer in men) and 8390 deaths in 2014 [1]. This problem is even more pronounced on the global scale, with 442,760 incident cases and 241,458 deaths worldwide in 2012 [2]; rates are particularly high in parts of Western Europe, Southeast Asia, and Oceania. The relatively high mortality is, in part, due to the fact that the majority of patients initially present at an advanced stage [3], which is associated with a much poorer prognosis [4]. Additionally, oral and pharyngeal cancer carries a very high morbidity, often with disfigurement and impairment of basic functions, such as talking, swallowing, eating, and breathing [3], that is exacerbated by more advanced disease and the associated disease treatments. Taken together, these considerations underscore the critical importance of early detection in reducing the adverse impact of this disease.

DNA methylation is a very common epigenetic event associated with the genesis of oral and pharyngeal carcinoma, often preceding the onset of frank malignancy [5]. DNA methylation occurs primarily in the context of CpG dinucleotides [6], which are disproportionately concentrated in enriched regions referred to as CpG islands. CpG islands are commonly situated in the 5′ promoter region of genes where their methylation is generally associated with transcriptional repression. However, methylation of CpG islands situated in inter- and intra-genic enhancer regions can also impact the timing or spatial patterns of gene expression [7]; there is mounting evidence that methylation of CpG islands located in the gene body can lead to *increased* transcriptional activation [8, 9]. Furthermore, regional methylation can impact the expression of non-coding RNA [7], the sequences of which are commonly situated in intronic or intergenic regions. Methylation of CpG islands can arise aberrantly during disease development and progression [6] but can also occur as part of normal biological processes, such as X-inactivation, imprinting [5], or tissue differentiation [10–14].

Currently, no proven screening techniques are in widespread use for oral and pharyngeal cancer aside from visual inspection and palpation, which are provided by dentists and clinicians on an opportunistic basis, lack sensitivity (particularly for pharyngeal tumors), and vary according to the skill of the clinician performing the exam. Oral rinse can be utilized as a non-invasive ascertainment technique for detection of DNA methylation in these cancers [15–27] and therefore has potential in biomarker-based screening applications, particularly among high-risk groups or for post-treatment surveillance. While the existing literature has primarily focused on a limited set of candidate promoter regions, epigenome-wide strategies offer a more comprehensive approach for discovery. Systematic evaluation of methylation over predefined aggregate regions, such as CpG islands, can help to mitigate issues relating to false discovery rate and technical noise that can complicate epigenome-wide assessment of large numbers of individual loci [28]. Hence, the goal of this study was to begin to address these needs through epigenome-wide interrogation via the Infinium HumanMethylation450 BeadArray for identification and validation of a novel sentinel CpG island methylation profile in non-invasive oral rinse samples that may be useful in predicting oral and pharyngeal carcinoma.

## Results

**Fig. 1:**
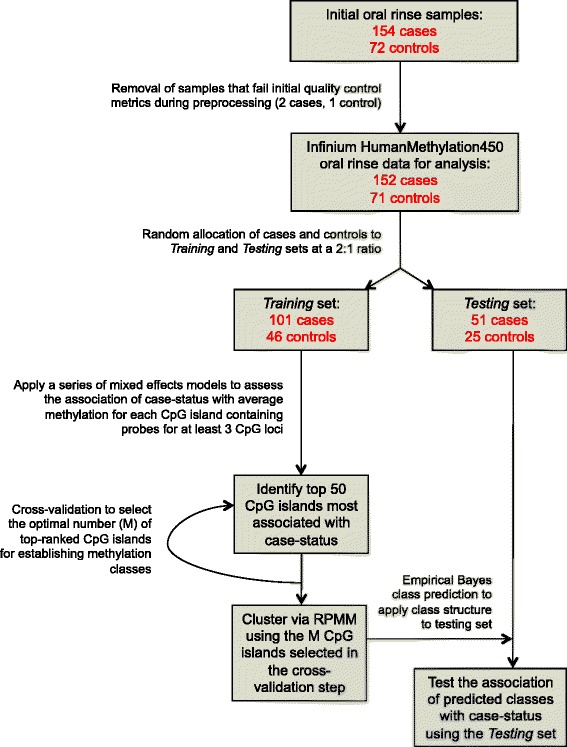
Schematic of the SS-RPMM algorithm for identification of a CpG island methylation profile predictive of oral and pharyngeal carcinoma case status

The study population included 154 cases with incident initial primary oral or pharyngeal squamous cell carcinoma from the greater Boston area and 72 cancer-free controls, from whom oral rinse samples were obtained. DNA methylation was interrogated in the oral rinse samples using the Infinium HumanMethylation450 BeadArray (Illumina, San Diego, CA), which contains probes for more than 450,000 CpG loci across 99 % of annotated human genes. A general schematic of our analytic workflow is presented in Fig. 1. Study participants were randomly partitioned into either a *training* or *testing* set at a 2:1 ratio, which resulted in 157 subjects in the *training* set (101 cases, 46 controls) and 76 in the *testing* set (51 cases, 25 controls); 3 of the original 226 samples (2 cases, 1 control) failed initial quality control measures and were excluded from the analyses. A description of the study population for the *training* and *testing* sets by case-control status is presented in Table 1.

**Table 1:** Characteristics of study subjects in the *training* and *testing* sets according to case-control status for oral and pharyngeal carcinoma

	Training set (*n* = 147)			Testing set (*n* = 76)		
	Case (*n* = 101)	Control (*n* = 46)	*p*_difference_	Case (*n* = 51)	Control (*n* = 25)	*p*_difference_
Age, median (range)	60.0 (23–86)	60.5 (46–88)	0.31^e^	58.0 (29–78)	59.0 (32–77)	0.82^e^
Sex						
Female	29 (28.7 %)	17 (37.0 %)	0.34^f^	16 (31.4 %)	8 (32.0 %)	>0.99^f^
Male	72 (71.3 %)	29 (63.0 %)		35 (68.6 %)	17 (68.0 %)	
Race^a^						
White	92 (91.1 %)	44 (95.7 %)	0.51^f^	47 (92.2 %)	20 (80.0 %)	0.15^f^
Other	8 (7.9 %)	2 (4.3 %)		4 (7.8 %)	5 (20.0 %)	
Smoking^b^						
Never	30 (31.3 %)	15 (32.6 %)	>0.99^f^	15 (31.3 %)	14 (56.0 %)	0.048^f^
Ever	66 (68.8 %)	31 (67.4 %)		33 (68.8 %)	11 (44.0 %)	
Pack-years^c^, median (range)	31.0 (0.6–120)	24.0 (0.1–200)	0.18^e^	22.0 (0.9–94)	13.7 (0.8–62.5)	0.23^e^
Alcohol use^b^						
Non-drinker	7 (7.4 %)	7 (15.2 %)	0.18^f^	3 (6.3 %)	4 (16.0 %)	0.047^f^
≤2 drinks/day	51 (53.7 %)	27 (58.7 %)		25 (52.1 %)	17 (68.0 %)	
>2 drinks/day	37 (38.9 %)	12 (26.1 %)		20 (41.7 %)	4 (16.0 %)	
HPV serology^d^ (E6 or E7 antibodies)						
Negative	62 (66.0 %)	–	–	26 (60.5 %)	–	–
Positive	32 (34.0 %)	–		17 (39.5 %)	–	
AJCC stage group						
I	24 (23.8 %)	–	–	13 (25.5 %)	–	–
II	12 (11.9 %)	–		7 (13.7 %)	–	
III	13 (12.9 %)	–		5 (9.8 %)	–	
IV	52 (51.5 %)	–		26 (51.0 %)	–	

^a^Race data was missing for 1 case in the *training* set

^b^Smoking and alcohol data were missing for 5 cases in the *training* set and 3 cases in the *testing* set

^c^Restricted to ever-smokers

^d^HPV16 E6 and/or E7 serology was missing for 7 cases in the *training* set and 8 cases in the *testing* set

^e^Wilcoxon rank-sum test

^f^Fisher’s exact test

There were a total of 32,465 autosomal CpG islands represented by at least three CpG probes on the HumanMethylation450 BeadArray. After fitting a series of individual linear mixed-effects models for average methylation across each of these CpG islands and ranking them according to absolute t-statistic using only the *training* set data, we used a semi-supervised recursively partitioned mixture modeling (SS-RPMM) algorithm [29], through which we determined that a methylation classifier based on 22 CpG islands formed the optimal number of top CpG loci for discriminating between cases and controls, resulting in seven distinct methylation classes. A description of each of these 22 CpG islands, along with their bioinformatic attributes, is presented in Table 2.

**Table 2:** Description of the 22 CpG islands used to establish the methylation classifier in oral rinse samples

M	CpG island coordinates^a^	Number of CpGs covered by the array	Associated gene (ncRNA)	CpG island relationship to gene	Enhancer region	DNase hypersensitivity site	Associated gene function^e^	Differential methylation in oral/pharyngeal tumor tissue^f^	
								FDR-adjusted Q-value	Median difference^g^ (range)
1	chr3:15286143-15286274	3	*SH3BP5*	5′UTR: body	True	True	Inhibits phosphorylation activity of Bruton Agammaglobulinemia Tyrosine Kinase; may play a role in BCR-induced apoptosis	1.12E−07	−0.19 (−0.35, −0.04)
2	chr17:77848690-77848800	3	(*JD529337*)^b^		True	True		9.86E−09	−0.14 (−0.17, −0.07)
3	chr12:118725604-118725889	3	*CIT*	Body	True		Serine/threonine-protein kinase that plays a role in cell division/cytokinesis	3.04E−09	0.32 (0.13, 0.46)
4	chr1:154198084-154198623	3	*ARHGEF2*	Body	True	True	Plays a fundamental role in cellular processes initiated by extracellular stimuli via G protein coupled receptors	4.21E−09	−0.22 (−0.31, −0.01)
5	chr12:28015205-28015607	3	*PTHLH*	5′UTR (TSS1500)			Neuroendocrine peptide member of the parathyroid hormone family that is a critical regulator of cellular and organ growth, development, migration, differentiation, survival, and epithelial calcium ion transport	3.97E−06	−0.19 (−0.29, 0.37)
6	chr11:2511670-2512178	4	*KCNQ1*	Body	True		Voltage-gated potassium channel required for the repolarization phase of the cardiac action potential; exhibits tissue-specific imprinting	3.04E−09	−0.29 (−0.52, −0.10)
7	chr1:8194584-8194818	3	(*JD505160*)		True	True		1.40E−06	−0.13 (−0.26, 0.09)
8	chr12:110319267-110319654	4	(see footnote)^c^					1.12E−07	−0.04 (−0.06, 0.01)
9	chr5:161207831-161208167	4	*GABRA1*	5′UTR (TSS1500:TSS200)			Receptor for gamma-aminobutyric acid (GABA), which is the major inhibitory neurotransmitter in the brain	5.08E−06	0.09 (−0.03, 0.66)
10	chr19:5538686-5538939	3	*SAFB2*	Body			Binds to scaffold/matrix attachment region (S/MAR) DNA; may function as an estrogen receptor corepressor or inhibitor of cell proliferation	0.00015	0.25 (−0.35, 0.27)
11	chr6:25135475-25135786	3	*BC070382* ^d^					3.04E−09	−0.29 (−0.47, −0.04)
12	chr10:134072408-134072501	3	*PWWP2B*	Body: 3′UTR			PWWP Domain-Containing Protein 2B	6.28E−08	0.21 (−0.10, 0.29)
13	chr1:10818517-10818704	3			True	True		0.00066	−0.16 (−0.28, 0.19)
14	chr1:1385949-1386143	5	*ATAD3C*	Body			ATPase Family AAA Domain-Containing Protein 3C	9.23E−08	−0.17 (−0.36, 0.03)
15	chr10:53743705-53744974	7	*DKK1*	5′UTR: Body		TRUE	Member of the dickkopf protein-coding gene family, which play an important role in vertebrate development	0.59	−0.02 (−0.10, 0.57)
16	chr11:20588323-20588561	3	*SLC6A5*	Body	True		Solute-carrier transporter involved in the clearance of extracellular glycine during glycine-mediated neurotransmission	3.51E−06	−0.03 (−0.06, 0.001)
17	chr10:134210902-134211265	5	*INPP5A*	Body			Membrane-associated type I inositol 1,4,5-trisphosphate (InsP3) 5-phosphate that mobilizes intracellular calcium and acts as a second messenger for mediating cell responses to various stimuli	1.89E−07	−0.09 (−0.42, −0.0004)
18	chr5:10702368-10703458	3	*ANKRD33B*	Body			Ankyrin Repeat Domain-Containing Protein 33B	7.67E−09	0.14 (0.03, 0.18)
19	chr16:85998896-85999172	3	*ZCCHC14*	3′UTR		TRUE	Zinc Finger CCHC Domain-Containing Protein 14; interacts with nuclear transcription factors NFIC and NFIX	0.13	0.09 (−0.42, 0.20)
20	chr13:105827274-105827476	3	(*LINC00460*)				(long non-coding RNA of unknown function)	3.34E−08	−0.26 (−0.48, 0.04)
21	chr5:1010475-1010610	3			True	True		8.09E−05	−0.19 (−0.34, 0.11)
22	chr2:216945117-216945376	6	*MARCH4*	5′UTR (TSS1 500:TSS200)			E3 ubiquitin-protein ligase that may mediate ubiquitination of MHC-I and CD4, and promote their subsequent endocytosis and sorting to lysosomes via multivesicular bodies	0.00076	−0.04 (−0.06, 0.08)

Abbreviations: *M* rank order of top CpG islands comprising the oral rinse methylation classifier, *UTR* untranslated region, *kb* kilobase, *TSS200* within 200 bases of transcription start site, *TSS1500* within 1500 bases of transcription start site, *ncRNA* non-coding RNA, *FDR* false discovery rate (Benjamini and Hochberg)

^a^Coordinates correspond to CpG islands predicted by Hidden Markov Model (HMM) using the NCBI36/hg18 assembly

^b^CpG island is <2 kb downstream of a bioinformatically detectable short RNA sequence

^c^CpG island is <1 kb upstream (JD366788, JD497927, JD365992) and downstream (JD358111, JD476820, JD415033) of several bioinformatically detectable short RNA sequences

^d^Hypothetical short protein-coding sequence

^e^Gene function was extracted from GeneCards (www.genecards.org)

^f^Based on Infinium HumanMethylation450 data from 34 tumor/matched-adjacent normal tissue pairs from The Cancer Genome Atlas (TCGA)

^g^Median difference in beta value of tumors relative to controls (positive value denotes relative hypermethylation; negative value denotes relative hypomethylation)

**Fig. 2:**
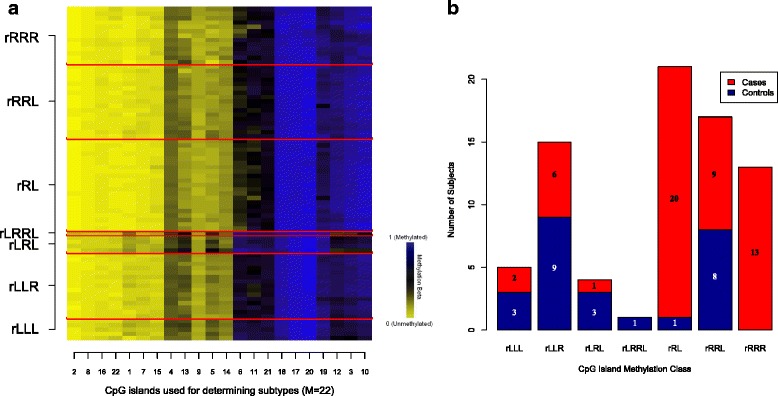
**a** Heatmap showing average methylation of the 22 CpG islands comprising the classifier for each of the 76 *testing* set subjects, clustered according to methylation class. The numbers in the *x*-axis correspond to the rank-ordered *M* number displayed for each respective CpG island in Table 2. **b** The distribution of cases (*n* = 51) and controls (*n* = 25) from the *testing* set across the seven methylation classes

We then validated this class structure and tested its predictive power by applying the latent structure of the methylation classes established in the *training* set to the *testing* set. A heatmap of the methylation profiles of each of the 22 CpG islands for the subjects assigned to the *testing* set by methylation class is presented in Fig. 2a. Two methylation classes, rRL and rRRR (denoted according to left and right branches on the dendogram from the clustering procedure), particularly stand out as being “case-heavy” (Fig. 2b), which collectively include 33 cases and only one control. When considered together, the association of these two classes with case status relative to all other classes is remarkably strong, with a crude odds ratio (OR) = 43.8 (95 % CI 8.1, 816.7) and adjusted OR = 76.9 (95 % CI 11.8, 1818.2). The sensitivity and specificity of classes rRL and rRRR for correctly predicting case status is 64.7 and 96.0 %, respectively.

**Fig. 3:**
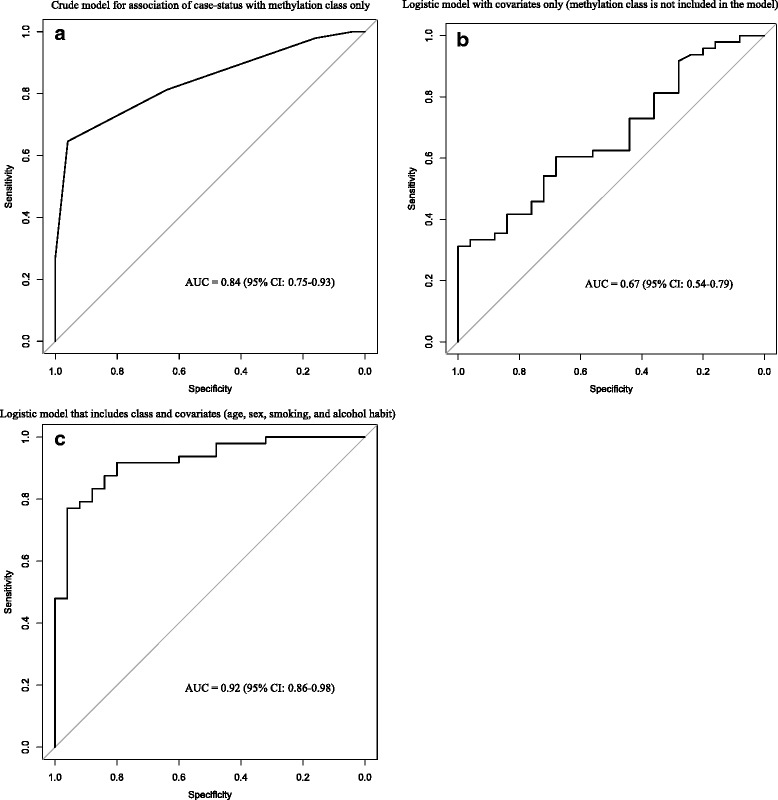
Receiver operator characteristic (ROC) curves with corresponding area under the curve (AUC) and 95 % confidence intervals (95 % CI) for logit regression for the association of oral and pharyngeal carcinoma case status in the *testing* set with **a** methylation class only; **b** age, sex, smoking pack-years, and alcohol consumption only; and **c** methylation class adjusted for age, sex, smoking pack-years, and alcohol consumption

To further assess the performance of the classifier in the *testing* set, we constructed receiver operating characteristic (ROC) curves for the logistic regression models and calculated the corresponding area under the curve (AUC) (Fig. 3). When considering the association between case status and methylation class alone (i.e., no other independent covariates in the model), the AUC was 0.84 (95 % CI 0.75–0.93). After additional adjustment for age, sex, smoking pack-years, and alcohol consumption, the AUC increased to 0.92 (95 % CI 0.86, 0.98).

In an effort to explore possible drivers of these findings, we assessed the sociodemographic and clinical characteristics of oral and pharyngeal carcinoma cases for each “case” class (rRL, *n* = 20; rRRR, *n* = 13) relative to cases in all other classes (*n* = 18) (Table 3). No significant differences were observed across classes. However, while non-significant, class rRRR had a higher fraction of cases with seropositivity for HPV16 e6/e7 antibodies (*p* = 0.11), which were performed on study subjects as a biomarker of HPV16-transformed invasive tumors [30]. Additionally, the case classes contained a somewhat higher, albeit non-significant, proportion of advanced stage cases, although it is notable that both of these cases also contain a sizable fraction of localized cancers and that the majority of cases have relatively smaller (T1–T2) tumors.

**Table 3:** Characteristics of *testing* set cases for each of the two case-associated methylation classes (rRL and rRRR) relative to those in the other five classes

	Methylation class			
Characteristic	rRL (*n* = 20)	rRRR (*n* = 13)	All other classes (*n* = 18)	*p*_difference_
Age, median (range)	56.5 (29–78)	54.0 (33–78)	62.5 (31–76)	0.43^c^
Sex				
Female	8 (40.0 %)	2 (15.4 %)	6 (33.3 %)	0.33^d^
Male	12 (60.0 %)	11 (84.6 %)	12 (66.7 %)	
Race				
White	18 (90.0 %)	11 (84.6 %)	18 (100 %)	0.29^d^
Other	2 (10.0 %)	2 (15.4 %)	0	
Cigarette smoking^a^				
Never	7 (38.9 %)	4 (30.8 %)	4 (23.5 %)	0.63^d^
Ever	11 (61.1 %)	9 (69.2 %)	13 (76.5 %)	
Pack-years, median (range)	16.5 (1.2–48)	25.0 (5.5–60)	22.0 (0.9–94)	0.56^c^
Alcohol use^a^				
Non-drinker	1 (5.6 %)	2 (15.4 %)	0	0.21^d^
≤2 drinks/day	10 (55.6 %)	8 (61.5 %)	7 (41.2 %)	
>2 drinks/day	7 (38.9 %)	3 (23.1 %)	10 (58.8 %)	
HPV serology^b^ (E6 or E7 antibodies)				
Negative	12 (66.7 %)	4 (36.4 %)	13 (76.5 %)	0.11^d^
Positive	6 (33.3 %)	7 (63.6 %)	4 (23.5 %)	
Primary tumor site				
Oral cavity	13 (65.0 %)	6 (46.2 %)	10 (55.6 %)	0.53^d^
Oropharynx	4 (20.0 %)	6 (46.2 %)	7 (38.9 %)	
Hypopharynx	3 (15.0 %)	1 (7.7 %)	1 (5.6 %)	
AJCC stage group				
Local (stage I or II)	6 (30.0 %)	4 (30.8 %)	10 (55.6 %)	0.25^d^
Advanced (stage III or IV)	14 (70.0 %)	9 (69.2 %)	8 (44.4 %)	
Tumor size (T class)				
T1–T2	14 (70.0 %)	9 (69.2 %)	16 (88.9 %)	0.29^d^
T3–T4	6 (30.0 %)	4 (30.8 %)	2 (11.1 %)	

^a^Smoking and alcohol data were missing for 2 cases in class rRL and 1 case in “All other classes”

^b^HPV16 E6/E7 serology data was missing for 2 cases in class rRRR and 1 case in “All other classes”

^c^Kruskall-Wallis test

^d^Fisher’s exact test

To evaluate the biological significance of the 22 CpG islands that form the oral rinse methylation classifier, we downloaded Infinium HumanMethylation450 BeadArray data from The Cancer Genome Atlas (TCGA; http://cancergenome.nih.gov/) for all oral and pharyngeal carcinoma cases with paired adjacent normal tissue (34 pairs). With respect to the CpG islands forming the classifier, 20 of 22 were found to be significantly differentially methylated in tumor tissue relative to the adjacent normal tissue, based on the Wilcoxon signed-rank test and adjusting for false discovery rate (FDR) using the methods of Benjamini and Hochberg [31]. The results of this analysis are presented in the right-hand columns of Table 2. Of note, the two CpG islands that were not significantly differentially methylated exhibited broad variability from the minimum to maximum differentials, which could potentially add information on a subset of samples to the classifier, particularly when considered in conjunction with other CpG islands.

## Discussion

We have identified a CpG island methylation classifier that can be used with oral rinse samples for predicting incident oral and pharyngeal carcinoma with a high degree of accuracy. Several other studies have examined the potential utility of DNA methylation in oral rinse samples for predicting head and neck cancers [15–25], but our findings represent the strongest predictive panel reported to date that was validated in an independent study set, with an impressive adjusted AUC of 0.92 (several studies reporting high sensitivity and/or specificity established methylation cut-points using the same set of samples that predictive accuracy was tested, leaving them susceptible to issues from over-fitting). By applying a rigorous two-stage analysis of Infinium HumanMethylation450 BeadArray data with an agnostic genome-wide assessment that encompassed all annotated CpG islands, including those outside of the gene promoter context, our study provides contrast to the majority of existing studies, which with few exceptions employ a candidate-gene approach centered on promoter methylation. As such, this study has given rise to novel targets, the majority of which, to our knowledge, have not been previously reported.

Adding further strength to our findings, 20 of the 22 CpG islands were observed to be differentially methylated in tumor tissue relative to adjacent normal tissue. One of the two CpG islands that was not differentially methylated in the TCGA tumors overlaps the promoter region of *DKK1*, which has been reported to be hypermethylated in head and neck carcinoma [32, 33], and lower expression has been associated with increased risk of lymph node metastasis and poorer outcome [34, 35], although other studies report conflicting findings [36, 37]. The other CpG island that was not differentially methylated in the TCGA tumors is associated with the zinc-finger protein *ZCCHC14*, which is an intriguing locus, as SNPs in that gene have been associated with nicotine dependence [38]. The CpG island is located in the 3′UTR of *ZCCHC14* and overlaps putative microRNA-binding sites for miR-542-3p and miR-615-3p [39].

The strengths of this study include the relatively large number of cases and controls with oral rinse samples compared to the majority of studies in the current body of literature; the use of average CpG island methylation in conjunction with the broad coverage from Infinium HumanMethylation450 BeadArray helps to mitigate technical noise that is often an impediment with single locus analysis; and the inclusion of oropharyngeal and hypopharyngeal cases along with oral cavity cases broadens the potential applicability of this panel. Additionally, our agnostic, data-driven approach with the inclusion of all CpG islands rather than select, candidate promoter methylation can also be viewed as a strength. This does not diminish the importance of the candidate genes used in prior studies, but we have now added novel CpG island loci for investigation in future studies. One potential weakness of this study is its retrospective design, although, conversely, the case-control design has the advantage of providing us with a much larger number of oral and pharyngeal carcinoma cases than we would be able to obtain using a population-based prospective study. Future studies will be aimed at prospective validation the 22 CpG island methylation panel as a screening tool in a high-risk population and as a potential tool for use in post-treatment follow-up surveillance for head and neck cancer patients. Additionally, due to coverage limitations of the Infinium HumanMethylation450 BeadArray, it is plausible that we may have missed some CpG islands that could potentially play an important epigenetic role in oral and pharyngeal carcinoma by restricting our analysis to CpG islands containing at least three CpG probes on the array. However, this platform offers excellent coverage of CpG-dense regions, allowing us to analyze >32,000 distinct CpG islands (as defined by Hidden Markov Model), and remains among the best available options for epigenome-wide analysis of a large number of samples [40–42].

## Conclusions

Although further expanded testing is warranted in a prospective setting, this panel may have utility for early detection of disease, particularly in targeted, high-risk populations. Importantly, methylation panels used in conjunction with non-invasive oral rinse samples, such as that described herein, may ultimately prove valuable as an aid for post-treatment follow-up surveillance; again, further prospective testing of this methylation classifier is warranted to determine its applicability to such applications. Continued discovery and development of clinically relevant biomarkers that can help with early detection of incident and/or recurrent head and neck cancer will ultimately have a positive impact on public health by reducing morbidity and mortality associated with this devastating disease, both in the USA and worldwide.

## Methods

### Study population

The study population included 154 cases with incident initial primary squamous cell carcinoma arising in the oral cavity (ICD-9: 141.1–141.5, 141.8, 141.9, 143–145.2, 145.5–145.9, 149.8, 149.9), oropharynx (ICD-9: 141.0, 141.6, 145.3, 145.4, 146, 149.0, 149.1), or hypopharynx (ICD-9: 148) diagnosed between October 2006 and June 2011 at major teaching hospitals located in Boston, MA (Brigham and Women’s Hospital, Beth Israel Deaconess Medical Center, Boston Medical Center, Dana-Farber Cancer Institute, Massachusetts Eye and Ear Infirmary, Massachusetts General Hospital, and New England Medical Center) as part of a population-based study of head and neck cancer in the greater Boston area (Collaborative Study of Head and Neck Diseases (CoHANDS)) that has been previously described [43, 44]. For inclusion in the study, cases were required to reside in the greater Boston area or any of 162 contiguous cities and towns within an approximately 1-h drive from Boston at the time of diagnosis. Cases with a prior history of malignancy other than non-melanoma skin cancer were excluded from the analyses. Cancer-free control subjects (*n* = 72) were randomly selected from 567 controls that were recruited into CoHANDS using a population-based design [45] during the same time frame as the cases. All patients included in the analyses provided written informed consent prior to enrollment in the study, as approved by the institutional review boards of Brown University and the participating institutions listed above.

### Sample collection, DNA extraction, and bisulfite modification

Upon enrollment into CoHANDS (and prior to initiation of treatment for cases), subjects were asked to vigorously swish with approximately 30 ml of commercial alcohol-free mouthwash (Act™) for 30 s. Samples were then centrifuged into cell pellets and stored at −80 °C in cryovials until DNA extraction. DNA was extracted using the QIAamp Blood Kit (Qiagen, Valencia, CA) using the spin protocol for DNA purification from blood or body fluids. Extracted DNA was bisulfite modified using the EZ-96 DNA Methylation-Direct Kit (Zymo Research, Irvine, CA) according to Illumina’s recommendations for the Infinium HumanMethylation450 BeadArray.

### Infinium HumanMethylation450 BeadArray

The Infinium HumanMethylation450 BeadArray assay was performed in three batches at the University of California San Francisco (UCSF) Institute for Human Genomics Core Facility (first and second batches) and University of Cincinnati (UC) Genomics, Epigenomics and Sequencing Core (third batch). Approximately 500 ng of bisulfite-modified genomic DNA was provided to the respective facility for initial processing of the BeadArrays, with samples randomized to BeadChip positions to mitigate any impact of potential batch or chip effects. Raw image files were preprocessed using the RnBeads pipeline in R [46]. All array data points are represented by fluorescent signals from both methylated (Cy5) and unmethylated (Cy3) alleles, and average methylation level (β) is derived from the ~18 replicate methylation measurements, β = (max(Cy5, 0))/(|Cy3| + |Cy5| + 100). Beta (β) = 1 indicates complete methylation; β = 0 represents no methylation. Outliers were assessed using quality control plots generated through the RnBeads pipeline designed to diagnose problems such as poor bisulfite conversion or signal intensity issues. Functional normalization was performed using *minfi.funnorm* [47] following background correction with the *normal-exponential using out-of-band probes* (NOOB) method [48]. Any probes with a detection *p* value >0.01 or that contained a single-nucleotide polymorphism (SNP) in the probe sequence were filtered out of the dataset prior to analysis. To account for any residual batch or chip effects, methylation data were adjusted using the ComBat method [49] via the SVA package in Bioconductor. The dataset supporting the results of this article are available the Gene Expression Omnibus (GSE7097: http://www.ncbi.nlm.nih.gov/geo/query/acc.cgi?acc=GSE70977).

### Statistical analysis

Average methylation was calculated for each autosomal CpG island (determined by the Hidden Markov Model approach, which provides a more accurate approach for identification of CpG islands than expected CG content-based filtering strategies [50]) that spanned at least three CpG loci on the HumanMethylation450 BeadArray (after filtering). It was our intent that assessment of CpG island methylation (as opposed to the individual locus approach) would provide more stable estimates that are less readily influenced by outliers due to technical variation.

A semi-supervised recursively partitioned mixture modeling (SS-RPMM) algorithm [29] was applied to identify a novel set of CpG islands for which methylation in oral rinse samples were predictive of oral and pharyngeal carcinoma case status. This method is based both on the semi-supervised procedure proposed by Bair and Tibshirani [51, 52] and recursively partitioned mixture models (RPMM) developed by Houseman et al. [53]. To avoid over-fitting the data and provide for validation of the model, subjects were randomly partitioned into either a *training* set (for the initial analysis) or a *testing* set (for subsequent validation) at a 2:1 ratio (frontloaded to increase the precision of the classifier identified in the *training* set), stratified by case-control status to ensure an equal distribution between sets. A series of linear mixed-effects models were then fit to logit-transformed average methylation (M) values to identify CpG islands most associated with case status, and were adjusted for age, sex, smoking pack-years, and alcohol consumption (typical number of alcoholic beverages per week) with a random-effect term for batch/processing site (UCSF or UC). CpG islands were ranked based on the absolute value of the t-statistic for case status. The top *M* loci were selected using a nested cross-validation procedure to train a classifier for case/control status by fitting a RPMM to the training data to cluster subjects using the *M* selected loci. To predict class membership in the *testing* set, the latent class structure from the RPMM fit to the training data was applied using an empirical Bayes procedure. Unconditional logistic regression was used to calculate the magnitude of the association between methylation class and oral and pharyngeal carcinoma, controlling for potential confounding covariates (age, sex, smoking pack-years, and alcohol consumption). Receiver operating characteristic (ROC) curves and corresponding area under the curve (AUC) were generated to assess the performance of the DNA methylation classifier.

## Abbreviations

AUC: area under the curve
CpG: cytosine-guanine dinucleotide
DNA: deoxyribonucleic acid
OR: odds ratio
RNA: ribonucleic acid
ROC: receiver operating characteristic
TCGA: The Cancer Genome Atlas
